# Clinical efficacy of Bone Cement-injectable Cannulated Pedicle Screw Short Segment Fixation for Lumbar Spondylolisthesis with Osteoporosise

**DOI:** 10.1038/s41598-020-60980-w

**Published:** 2020-03-03

**Authors:** Yao-yao Liu, Jun Xiao, Xiang Yin, Ming-yong Liu, Jian-hua Zhao, Peng Liu, Fei Dai

**Affiliations:** 1 Department of Spine surgery, Army Medical Center of PLA, Chongqing, 400042 People’s Republic of China; 2 Department of Orthopedics, Southwest Hospital of Army Medical University, PLA, Chongqing, 400038 People’s Republic of China

**Keywords:** Cell biology, Cell biology, Ocean sciences, Ocean sciences

## Abstract

Many clinical studies have shown a satisfactory clinical efficacy using bone cement-augmented pedicle screw in osteoporotic spine, however, few studies have involved the application of this type of screw in lumbar spondylolisthesis. This study aims to investigate the mid-term clinical outcome of bone cement-injectable cannulated pedicle screw (CICPS) in lumbar spondylolisthesis with osteoporosis. From 2011 to 2015, twenty-three patients with transforminal lumbar interbody fusion (TLIF) using CICPS for lumbar spondylolisthesis were enrolled in the study. Oswestry Disability Index (ODI) and Visual Analogue Scale (VAS) were used to evaluate faunctional recovery and physical pain; and operation time, blood loss and hospitalization time were recorded, respectively. Radiograph and computed tomography of lumbar spine was performed to assess loss of the intervertebral disc space height, fixation loosening, and the rate of bony fusion. The average follow-up time of 23 patients was 22.5 ± 10.2 months (range, 6–36 months). According to VAS and ODI scores, postoperative pain sensation and activity function were significantly improved (p < 0.05). The height of the intervertebral disc space was reduced by 0.4 ± 1.1 mm, and the bone graft fusion rate was 100%. No cases of internal fixation loosening or screw pullout was observed. CICPS using cement augmentation may suggest as a feasible surgical technique in osteoporotic patients with lumbar spondylolisthesis.

## Introduction

Lumbar spondylolisthesis is a common clinical spine disease. In elderly osteoporosis spine, the anti-pullout force of the pedicle screws is significantly inadequate owing to the poor bone quality. Intraoperative and postoperative screw loosening and other types of failure occurs frequently^1^. Screw failure during the operation leads to difficulties in vertebral body reduction, especially in lumbar spondylolisthesis. To solve this problem, operative segment needs to be extended or screw path augmentation needs to be carried out, which leads to the increase of operation time. However, the effect after augmentation is not accurate, screw failure may reoccur. Bony fusion failure observe during the postoperative rehabilitation process ultimately, which causes symptoms such as osphyalgia and sciatica. Therefore, improving the stability of internal fixation and increasing the bony fusion rate in lumbar spondylolisthesis with osteoporosis have always been the challenges facing spine surgeons.

**Figure 1:**
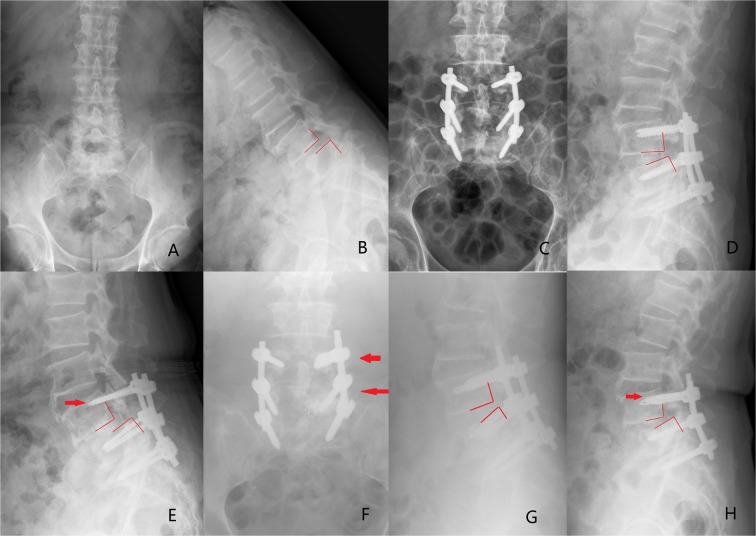
(**A,B**) A 74-year-old male diagnosed as spondylolysis at the L4 vertebral body with osteoporosis(T = −3.2, degree I). (**C,D**) Immediate postoperative radiographs showed reconstruction for spondylolisthesis compared with that before surgery. (**E**) Internal fixation failure occurred 1 month after surgery. Lateral radiograph showed L4 vertebral body was displaced again and the red arrow showed screw loosening and pullout. Patient redeveloped the lumbocrural pain. (**F,G**) After treatment with screw tunnel augmentation using PMMA (left side of L4 and L5, as the red arrows showed), L4 vertebral body was reset again. (**H**) One month after the revision surgery, however, screw failure occurred again (the red arrows showed the PMMA augmentation screw loosening).

Traditional methods to improve the stability of pedicle screw are as follows: (1) Increasing the length and diameter of the screws^2^; (2) Improving the screw design, such as expansion screw^3,4^; and (3) Using bone granules or bone cement such as polymethylmethacrylate (PMMA) to strengthen the screw tunnel^5–7^. Due to the poor bone quality in osteoporosis spine, most of the methods are not effective. Although PMMA augmentation is considered a reliable and feasible method up to the present time. However, screw tunnel augmentation with PMMA is a complicated procedure, which may also increase the operation time, the amount of bleeding and the risk of PMMA leakage (approximately 30%)^8^. Surgeons showed great anxiety about internal fixation failure especially in the treatment of lumbar spondylolisthesis with osteoporosis. Figure 1 shows a typical case of postoperative screw loosening in this condition. To overcome this problem, authors improved the design and created a new type of hollow cement-augmented pedicle screw (CAPS) named bone cement-injectable cannulated pedicle screw (CICPS).

To evaluate the results and feasibility of this technique, we retrospectively reviewed the data of 23 consecutive patients with lumbar spondylolisthesis treated with TLIF using CICPS, and summarized the incidence of complications and the clinical efficacy of PMMA augmentation.

## Materials and Methods

### Clinical data

#### CICPS design

CICPS was designed as a hollow screw rod with three side holes for bone cement outflow at the tip. The side holes were arranged from small to large. In this manner, bone cement could flow out of the side holes evenly to achieve a “Root”-like riveting strengthening effect. Less bone cement usage is needed in CICPS, which get benefit from the special side hole design. In refs. ^10,11^, we have described the design features of CICPS in detail.

### General data

From 2011 to 2015, consecutive 23 patients compriseing 9 males and 14 females with lumbar spondylolisthesis and osteoporosis were reviewed in the study. The average age at the time of surgery was 63.3 ± 7.8 years (rang, 56–79 y). Inclusion criteria were as follows: 1. Patient age >60 years; 2. Single level lumbar spondylolisthesis (X-ray); 3. T-score <−2.5 SD on dual-energy x-ray absorptiometry^9^; and 4. No surgical contraindications. Exclusion criteria were as follows: 1.Allergy to the implant; 2. Normal bone mineral density (BMD); 3. Presence of other spine diseases; and 4. Infections, blood system-related diseases, or other surgical contraindications. Osteoporosis was graded according to the Jikei grading scale. There were 13 cases with level II and 10 cases with level III^10^. According to the Meyerding classification of spondylolysis^11^, 10 cases had degree I, 9 cases had degree II, and 4 cases had degree III. General information of patients is presented in Table 1. The study was approved by the Daping Hospital ethics committee (IRB, 2019149). All methods were performed in accordance with the relevant guidelines and regulations. And all patients included in this study gave their informed consent.

**Table 1:** General information of patients.

Variable	Value
Average age	63.3 ± 7.8(56–79)
Gender (male: female)	9:14
Weight (kg)	48.5 ± 10.8
BMI (kg/m2)	19.0 ± 1.5
**Jikei BMD classification**	
Level II	13/23 (56.5%)
Level III	10/23 (43.5%)
**Meyerding classification of spondylolysis**	
Degree I	10/23 (43.5%)
Degree II	9/23 (39.1%)
Degree III	4/23 (17.4%)
Operation time (min)	145 ± 35 (85–220)
Blood loss (ml)	225 ± 87 (150–400)
Hospital stay (d)	7.9 ± 1.5(6–11)
**Complication**	
Superficial infection of the wound	1/6 (16.7%)
Bone cement leakage	4/6 (66.7%)
Cerebrospinal fluid leakage	1/6 (16.7%)
Fusion rate	100%
The number of conventional screws /total number of conventional screws	0/7
The number of loose CICPSs/total number of CICPSs	0/85

### Surgical method

TLIF or mis-TLIF was routinely performed. The screws were placed manually according to the anatomical landmarks. The angle of the CICPS was slightly larger than that of the conventional pedicle screw, which could reduced the risk of bone cement leakage^12,13^. Inaddition, the following attentions were taken to ensure the augmentation effect and further reduce bone cement leakage risk: 1. CICPS suggested to keep parallel to the upper and lower endplates in the process of screwing; 2. The probe was used to detect the required screw length so that the outflow side holes was located in the anterior 2/5^th^ of the vertebral body; and 3. CICPS was not allowed to penetrate the the pedicle wall and the lateral cortex of the vertebral body. In principle, we recommended a minimum number of CICPSs should be used^13^, because the revision might be very difficult. In this group, when manual findings during tapping indicated that the fixation stability might be insufficient, CICPS augmentation was used. The remaining screws adopted conventional ones.

PMMA powder and water agent were mixed together, and then, they were inserted into a special bone cement syringe. During the period of a dough-like mass viscosity, 1–2 ml of PMMA mixture was injected into the vertebral body through a special connecting device. The whole injection procedure was controlled by a C-arm machine; if PMMA leakage was observed, stop injecting immediately. A universal or single-axis screw head was adopted according to the requirements of installing rods. After the PMMA cement had completely hardened, rods were installed; and nuts were locked to reposition the slipped vertebral body. Then, routine spinal canal decompression and bone graft fusion were performed.

### Postoperative treatment

In this study, antibiotics were routinely used to prevent surgical site infection in the first 48 hours. Cardiopulmonary rehabilitation training in bed was allowed in the first 24 hours, and the suction drain should be removed within 3 days when the amount of drainage fluid was less than 50 ml. Three days after surgery, the patients was encouraged to get out of bed under the protection of thoracolumbar brace. The patients needed to bear the thoracolumbar brace continuously until 3 months after operation.

### Evaluation method

The operation data including operation time, blood loss, and hospitalization time in patients were recorded. To evaluate patients’ pain and functional recovery, VAS and ODI score were compared before and after surgery, respectively. Radiograph and computed tomography were used to determine pedicle screw loosening, loss of the intervertebral disc space height, and bony fusion rate. Perioperative complications, for instance, postoperative infection, PMMA and cerebrospinal fluid leakage were also recorded.

Evaluation criteria for screw loosening on an X-ray were as follows: (1) Screw displacement greater than 1 mm on a lateral radiograph^14,15^; and (2) Double-circle sign around the screw on an anterior radiograph^16^.

The method of measuring the height of the intervertebral disc space was as follows: On a lateral X-ray film, to measure the distance between the posterior edge of dislocated vertebra and the upper vertebral lamina of the lower vertebra, and that between the anterior edge of the lower vertebra and lower lamina of the dislocated vertebra. The average of these two value represented the height of the intervertebral disc space. And the difference between the heights of the intervertebral disc space before surgery and at the last follow-up was considered as the value for loss of the intervertebral disc space height.

Evaluation criteria for spinal fusion were as follows^17^: (1) Trabecular bone overgrew the graft area; (2) Vertebral body movement was <3 mm or the intervertebral angle change was <5 degree in the flexion-extension radiographs.

All data were measured using the picture archiving and communication systems (PACS) of the PLA Army Characteristic Medical Center. The experimental data were analyzed by using the SPSS 13.0 statistical software package. The measurement data were calculated as mean ± standard deviation (x ± s). VAS and ODI score, and imaging indicators were compared before surgery and final last follow-up using paired-t test. If p was less than 0.05, the difference was considered statistically significant.

### Ethics approval and consent to participate

This trial was approved by the ethical committe of Daping Hospital affiliated to Army Medical University, Chongqing, China (IRB number: 2019149).

## Results

All 23 patients were followed up for an average time period of 22.5 ± 10.2 months (range, 6–36 months). The average weight, height, and body mass index (BMI) were 48.5 ± 10.8 kg (range, 40.6–67.6 kg), 160.3 ± 6.3 cm (range, 148.2–175 cm), and 19.0 ± 1.5 kg/m^2^ (range, 18.4–21.4 kg/m^2^), respectively. The operation time was 145 ± 35 min (range, 85–220 min), the amount of bleeding was 225 ± 87 ml (range, 150–400 ml), and the hospitalization time was 7.9 ± 1.5 d (range, 6–11 d). Autologous blood transfusion was performed if necessary during the operation, and transfusion-related complications were not observed. However, One case of superficial surgical site infection was noted, and the infection was completely under control after an intravenous drip of antibiotics. One case of cerebrospinal fluid (CSF) leakage was observed. The drainage tube was removed after 1 week of bed rest, and then the incision healed completely.

A total of 85 CICPSs and 7 conventional screws were implanted. Each of the CICPSs were injected with approximately 1.0–2.0 ml of PMMA cement. Cement was not observed in the vertebral pedicle or spinal canal. No pulmonary embolism were detected in postoperative CT. Four patients developed cement leakage in the vertebral venous plexus without any neurological symptoms. There were no directly injuries to the nerves, blood vessels, and internal organs during the operation due to screw placement. During PMMA injection procedure, bone cement spillage at the device joint and surgical field contamination also did not occur. According to the criteria, no screw loosening was observed in all CICPSs and conventional screws. Fusion was achieved in all cases (Table 1).

**Figure 2:**
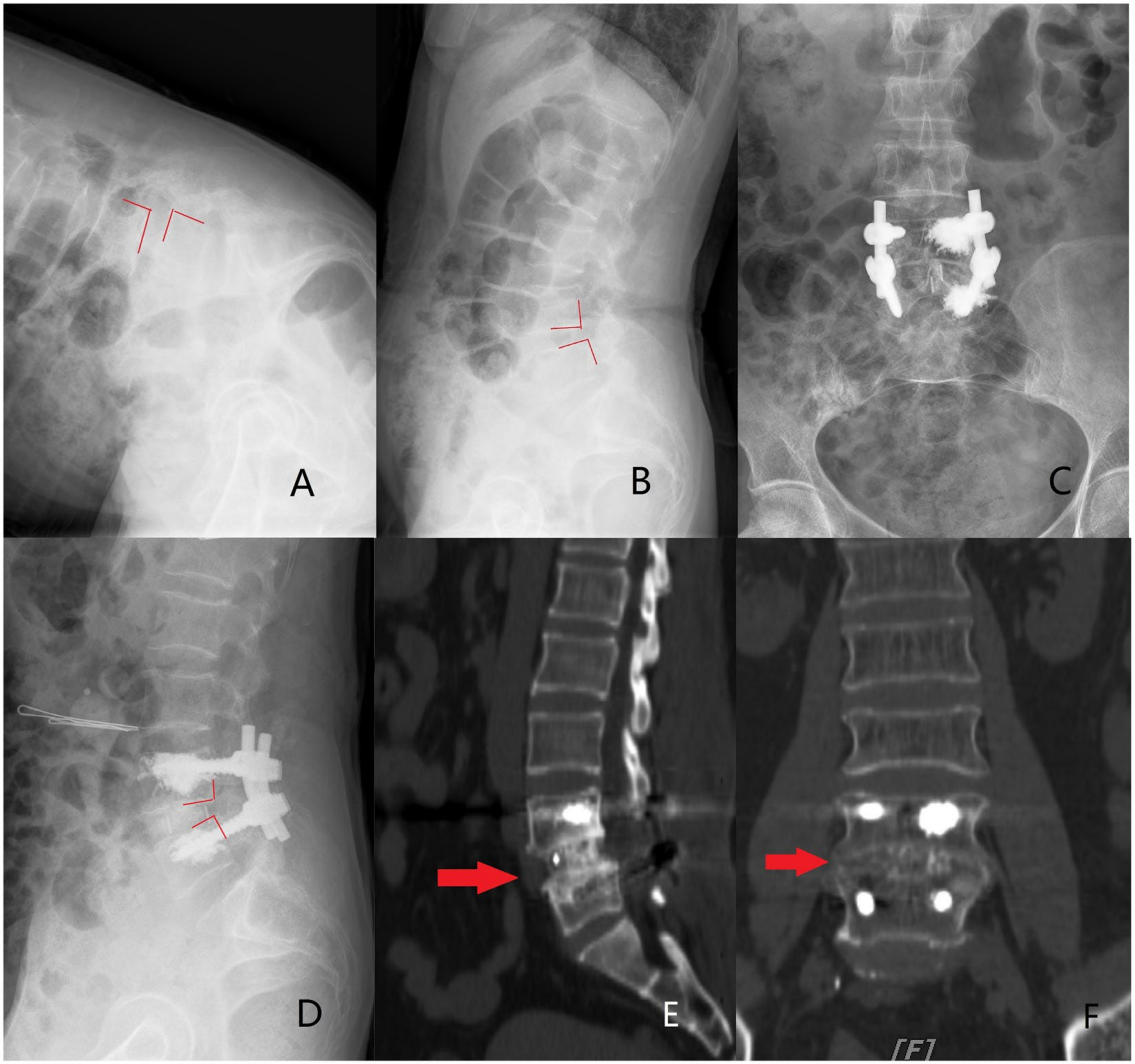
A 69-year-old female (T = −3.6) who was treated with unilateral 2 CICPSs fixation by PMMA augmentation. (**A,B**) Preoperative extension and flexion radiographs indicated the L4 vertebral body was unstable and displaced forward. PMMA seemed to leak to the anterior vertebral body. (**C,D**) The postoperative radiographs showed lumbar sequence reconstruction. (**E,F**) Three years after surgery, CT showed bony fusion achieved (the red arrow).

**Figure 3:**
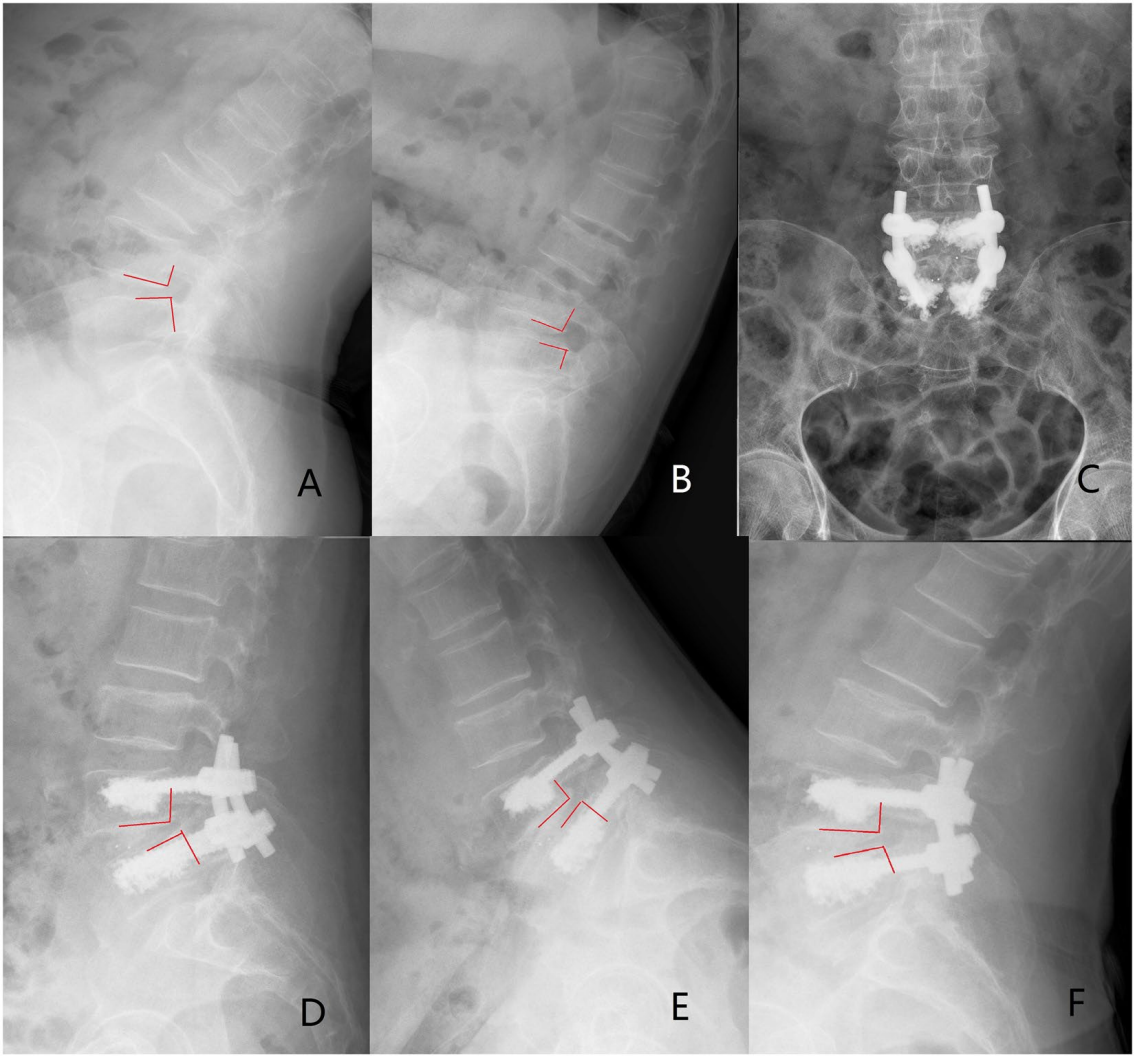
A 64-year-old female (T = −3.8) who was treated with bilateral 4 CICPSs fixation by PMMA augmentation. (**A,B**) Preoperative radiographs indicated the L4 spondylolisthesis. (**C,D**) The postoperative radiographs showed lumbar sequence reconstruction. (**E,F**) CICPSs was observed in place after 6 months of surgery and bony fusion was achieved.

The VAS and ODI scores were changed from 7.06 ± 2.67 points and 24.57 ± 10.63% preoperatively to 1.73 ± 0.81 points and 55.12 ± 28% at the last follow-up. Pain and dysfunction were improved in patients (VAS, p = 0.015, ODI, p = 0.003). The fusion rate of bone graft was 100%, and screw loosening was not observed. The intervertebral disc space height was 7.8 ± 3.7 mm before surgery, 10.1 ± 2.3 mm after surgery and 9.7 ± 1.2 mm at the last follow-up. This value was significantly increased after surgery compared with before surgery(p = 0.036). And the intervertebral disc space height was reduced by 0.4 ± 1.1 mm at the last follow-up compared with after surgery. There was no significant difference between these two periods (p = 0.62). The results are shown in Table 2 and the typical cases are shown in Figs. 2 and 3.

**Table 2:** The height of the intervertebral disc space, VAS score, and ODI score in patients before and after the treatment.

Index	Preoperative	Postoperative	Last follow-up
Intervertebral disc space height (mm)	7.8 ± 3.7	10.1 ± 2.3a	9.70 ± 1.2a,b
VAS score	7.06 ± 2.67	2.3 ± 1.31a	1.73 ± 0.81a,b
ODI score (%)	24.57 ± 10.63	50.3 ± 12.3a	55.12 ± 28a,b

^a^p < 0.05 vs. group Preoperative; ^b^p > 0.05 vs. group Postoperative.

## Discussion

Using a pedicle screw system to reconstruct the displaced vertebral body and perform bone fusion is still the main surgical method for patients in lumbar spondylolisthesis with osteoporosis^18^. Many studies have demonstrated the stability of pedicle screw is significantly reduced in osteoporotic spine with a screw loosening rate up to 12%^19,20^. Okuyama *et al*.^19^ reported that for every 10 mg/cm^2^ reduction in BMD, the maximum pullout force reduced by 60 N. When BMD was <80 to 90 mg/cm^2^, a pedicle screwcould not provide sufficient stability when special augmented method were required.

PMMA cement augmentation is considered a reliable and feasible method up to the present time. The traditional method is to inject PMMA into a preset screw tunnel, and then insert a screw. PMMA will disperses uncontrollably leading to a risk of leakage into the spinal canal, intervertebral foramen or vertebral venous plexus^21^. Moreover, PMMA releases a large amount of heat during the polymerization reaction. Neurologic damage will occur when PMMA closes to the spinal cord or nerve root^22^. More serious complications also could be observed such as pulmonary embolism^23^, paraplegia^24^, or death^25^.

To prevent this series of complications, hollow CAPS is considered an optional technique that has broad application prospects^26,27^. Chen *et al*.^28^ revealed the following findings: (1) Bone cement mainly flows out from the proximal side hole of CAPS, conversely, there is almost no cement outflow from the distal side hole; (2) In case of the same cement usage, the closer the proximal side hole to screw head, the more significant the increase of the pullout force but the higher the risk of cement leakage into spinal canal. Therefore, authors designed the side holes arranging from small to large on the anterior 2/5^th^ of the screw rod. This design may increase the cement amount outflow from the distal side hole, and then, evenly distributed in the vertebral body. By this method, the cement-leakage risk may be reduced in clinical application.

Revising experience of CAPS is barely reported up to now. It may be very difficult to remove the screws after cement hardened. Once screw loosening or wound infection in the restoration stage, patients will face a disastrous consequences. Therefore, the indications for CAPS using in patients needs further discussion. Based on the published literature, patients with vertebral osteoporosis is a necessary prerequisite for using CAPS in various studies, which is considered as a general consensus. The most commonly followed surgical indications include vertebral fractures, neurological deficit seriously decreasing quality of life, kyphosis with persistent symptoms and progressive kyphosis. In addition, Sawakami *et al*.^21^, Park *et al*.^29^ and Cho *et al*.^30^ had achieved satisfactorily clinical efficacy in the treatment of patients with pseudoarthrosis following Kummell’s lesion and vertebral fractures. So they considered secondary pseudarthrosis is one of the indications for CAPS augmentation. However, authors suggest the indications for using CICPS should include the following aspects: (1) Persistence low-back pain with aggravation; (2) Severe osteoporosis and conventional screw showed an inadequate holding force during the operation; (3) Progressive spondylolisthesis; (4) Symptoms and signs of cauda equina or nerve root; and (5) Patients with high weight.

Authors reviewed all the published literature pertaining to the use of CAPS in osteoporotic spine cases. Only three researches performed a controlled study relating to CAPS and noncement screws. El Saman *et al*.^31^ noted the loosening rate of CAPSs is 4.3%, whereas in noncement screws group, the loosening rate was 62.8%. Thus, a significantly lower loosening rate was showed in CAPSs compared to noncement ones. Sawakami *et al*.^21^ reported the screw loosening rate of CAPS was significantly lower than that of the control group (29.4% vs. 71.4%), meanwhile, a statistically significant increase was showed in fusion rate of CAPS group (94.1% vs. 76.1%). Patients’ back pain relieved obviously at follow-up (64.7%), and no PMMA-cement related complications was observed. Seo *et al*.^32^ reported two patients suffered from neurologic deficit due to PMMA leakage, but VAS and ODI was achieved a significant improvement in both cement and noncement groups. However, five cases of screw loosening occurred in the noncement group that further required a revision surgery for augmentation technology. Although, the first two studies used CAPS in patients with Kummell’s lesion and the last 1 in pseudoathrosis. We may conclude CAPS was possible to achieve satisfactory results in patients with osteoporosis.

Four clinical studies used CAPS in lumbar spondylolisthesis with osteoporosis were found. Pinera *et al*.^8^ investigated the clinical and radiological outcome of 23 patients with degenerative lumbar instability and osteoporosis treated with fusion using a type of CAPS. Pain and function improved at 6 months and this status was maintained at the final follow-up. The PMMA leakage rate was 29.3%. Three deep infections and one superfificial infection occured (17.4%). No clinical or radiological cases of non-fusion were observed. Jang *et al*.^33^ reported a comparative clinical studies. They evaluated 34 patients (17 patients with CAPS and 17 patients without cement augmentation) undergoing pedicle screw fixation for lumbar spondylolisthesis with osteoporosis. The results showed there was no difference in low back pain relief (VAS) between the two types of screw. No major perioperative complication in both groups. One patient (CAPS group, 5.9%) underwent revision surgery for the removal of a drainage tube, and one patient (noncement group, 5.9%) underwent revision surgery because of subcutaneous fluid collection. There was no events of radiological loosening, or pulling out of screws. Chandra *et al*. evaluated 25 patients separately underwent open-TLIF^34^ and minimally invasive-TLIF^35^ using CAPS fixation for lumbar spondylolisthesis. In the two studies, there was a statistically significant improvement in the VAS and ODI scores postoperatively. No events of radiological loosening, or pulling out of screws were observed. None of the patients had postoperative wound infection. Only one event of PMMA leakage was occured intraoperatively in open-TLIF operation (4%).

Authers used CICPSs in 23 patients with lumbar spondylolisthesis and osteoporosis. 4 patients (17.4%) were observed PMMA leakage, determined by X-ray film, without any neurological symptoms. In most studies, PMMA leakage presented no symptoms or only mild symptoms such as transient sensorimotor deficit. Janssen *et al*.^36^ reported that the incidence of asymptomatic PMMA leakage was 66.7% and that of symptoms was 5.5%. In symptomatic cases, 1.2% of the patients needed revision surgery to remove the screws and cement. Martín-Fernández *et al*.^37^ observed a 62.3% incidence of spinal leakage in 313 patients. 1.55% of the cases had symptoms which were presented as radicular pain of lower limbs. Lubansu *et al*.^38^ summarized some suggested tips to prevent bone cement leakage. However, up to now, the leakage rate of bone cement showed big difference^26,34,35^, which reason might be related to the small sample size or different screw design in different studies.

Pedicle screws with cement augmentation provided a strong holding force for the restruction of slipped vertebrae and maintain its stability to achieve interbody fusion. In the study, the height of intervertebral space maintained well during the follow up and all cases achieved bony fusion. Fixation loosening or backout was not observed in all CICPSs and no cases required revision. Similarly, the fusion rates were 100% in CAPS group compared with the noncement group in study by Sawakami *et al*.^21^. However, in control group, 3 (14.3%) cases occoured pedicle screw pullout and one of them required reoperation to stabilize the spine. Another 5 noncement cases needed to perform revision surgery reported by Seo *et al*.^32^. Martín-Fernández *et al*.^37^ reported the highest revision rate (17.9%) of the patients using CAPS. However, the revision reason for these 56 cases was not fixation loosening, but the adjacent segment-related issues such as pseudo-arthrosis formation and adjacent vertebral fracture.

Singh V *et al*.^39^ did a systematic analysis for cement-augmented screw. The results showed that the most commonly methods to evaluate faunctional recovery and physical pain were ODI and VAS scale. The average VAS score before surgery was 8.4 (range 8–9.2) compared to 2.3 (range 1.42–4.8) at the last follow up. The average improvement ODI for assessment of faunctional recovery was 42.1. Good to excellent clinical effect was reported in all these patients using CAPS. Vas and ODI are also used as evaluation indexes in our study. Last follow-up VAS was 1.73 compared with 7.06 preoperatively and ODI improved from 24.57 to 55.12. CICPS also showed a great improvement in pain relief and function recovery similar to these series.

Elderly patients, especially over 69 years old, were noticed a high incidence of complications with internal fixation treatment^40^. Singh V *et al*.^39^ reported 16 (1.5%) patients was observed superficial infection which treated with antibiotics; and 21 (2.1%) cases noted deep infection after surgery which were performed a debridement. The number of cement pulmonary embolism was 16 cases (1.5%). Cauda equina syndrome and cerebrospinal fluid leakage was noted in 1 patient and 3 ones, respectively. In our study, one case (8.6%) of superficial infection and one case (8.6%) of dural leak occurred. No overall cement pulmonary embolism and deep surgical site infection were observed. However, up to the present no enough evidence to prove CICPS reducing the risk of complications in elderly patients.

This study has several limitations. Firstly, the sample size is small and the follow-up time is short, thus a study with more patients and longer follow-up duration is needed to further confirm our results. Secondly, since the effectiveness of CICPS had been biomechanically evaluated before^13^, here only clinical evaluation was performed. A controlled randomized study could be suggested for further study. Finally, standard application protocol of CAPS should be developed to avoid the catastrophic consequences of excessive application, which will be our future research focus.

In summary, CICPS is a clinically safe and effective means which is used to augment pedicle screws in lumbar spondylolisthesis with osteoporosis. Up to present no enough evidence to recommend CAPS for osteoporotic spine. However, CICPS using PMMA augmentation may be considered a feasible surgical technique. This study could provide evidence-based basis for developing the guidelines of the CAPS application, especially in lumbar spondylolisthesis with osteoporosis.

## Data Availability

The datasets generated and analyzed during the present study are available from the corresponding author on reasonable request.
